# Microaxial flow pump in cardiogenic shock: a retrospective cohort study on outcomes and feasibility as a bridge to LVAD implantation

**DOI:** 10.1007/s12471-025-01963-2

**Published:** 2025-07-03

**Authors:** Jan-Willem Balder, Adriaan Kraaijeveld, Mariusz Szymanski, Linda van Laake, Einar Hart, Michiel Voskuil, Joep Droogh, Faiz Ramjankhan, Tim van de Hoef, Jeannine Hermens, Pim van der Harst, Kevin Damman, Erik Lipsic, Gabija Pundziute, Michael Dickinson

**Affiliations:** 1 Department of Cardiology, University Medical Centre Utrecht, Utrecht, The Netherlands; 2 Department of Intensive Care, University Medical Centre Groningen, Groningen, The Netherlands; 3 Department of Cardiothoracic Surgery, University Medical Centre Utrecht, Utrecht, The Netherlands; 4 Department of Intensive Care, University Medical Centre Utrecht, Utrecht, The Netherlands; 5 Department of Cardiology, University Medical Centre Groningen, Groningen, The Netherlands

**Keywords:** Cardiogenic shock, Mechanical circulatory support, Impella, Left ventricular assist device

## Abstract

**Introduction:**

In severe cardiogenic shock the use of temporary mechanical circulatory support has increased during the past decade, with the Impella CP emerging as an alternative to the intra-aortic balloon pump. This study aims to assess the clinical outcomes associated with Impella CP-only support in patients with cardiogenic shock within two tertiary institutions specialised in durable mechanical support in the Netherlands.

**Methods:**

All patients receiving Impella CP implantation for cardiogenic shock in the UMC Utrecht and UMC Groningen were studied between December 2020 and August 2023. Exclusion criteria included the use of additional mechanical support. The primary outcome evaluated was 30-day mortality. Secondary outcomes included LVAD implantations, cardiac recovery, and safety outcomes. Patients were categorised as Impella-optimal (Impella support expected to be sufficient) or Impella-limited (Impella support not sufficient but no further escalation because of comorbidities).

**Results:**

The cohort consisted of 28 patients. The mean age was 60 years, and 71% were males. In the Impella-limited group, mortality was 100%, and LVAD implantation was performed in just 10%, while in the Impella-optimal group, mortality was 17% and LVAD implantation was performed in 50%. Complications were primarily related to bleeding (7%) and vascular events (11%).

**Conclusions:**

This study suggests that Impella CP support in cardiogenic shock can be safe and feasible, serving as a bridge-to-recovery or a bridge-to-decision for LVAD implantation candidacy. However, Impella CP in the Impella-limited group is not useful. This underscores the importance of precise patient selection in cardiogenic shock therapy escalation.

**Supplementary Information:**

The online version of this article (10.1007/s12471-025-01963-2) contains supplementary material, which is available to authorized users.

## What’s new?

- Temporary mechanical circulatory support (tMCS) can be employed in cases of refractory cardiogenic shock to enhance tissue perfusion and oxygen delivery.
- tMCS has the potential to function either as a bridge-to-recovery or as a bridge-to-decision, involving the improvement of current organ function and the assessment of contra-indications for heart transplantation or the implantation of permanent left ventricular assist devices (LVADs).
- One type of tMCS is a percutaneously inserted intravascular microaxial pump (Impella CP).
- In our cohort, patients supported with Impella CP had a 30-day mortality of 46%.
- In the Impella-limited group (Impella support not sufficient but no further escalation because of comorbidities) mortality was 100% and LVAD implantation was performed in just 10%.
- In the Impella-optimal (Impella support expected to be sufficient) group mortality was 17% and LVAD implantation was performed in 50%.
- Patient selection in mechanical circulatory support is key to success.

## Introduction

In the setting of acute heart failure complicated by cardiogenic shock, the use of temporary mechanical support has increased significantly over the past decade [1]. Within the setting of acute myocardial infarction (MI) or high-risk percutaneous coronary intervention (PCI), the intra-aortic balloon pump (IABP) was formerly first choice. However, as the IABP-SHOCK II trial failed to indicate a survival benefit, IABP use has diminished over time [2, 3]. Currently, an alternative for temporary mechanical support is the microaxial left ventricle assist device, such as the Impella heart pump (Impella, Abiomed). This device is positioned across the aortic valve into the left ventricle and provides continuous antegrade flow, resulting in increased cardiac output, systemic perfusion and coronary flow, while reducing cardiac workload. The Impella 2.5 and Cardiac Power (CP) provide support up to 3.5 to 4.0 L/min whereas the 5.0 and 5.5 models provide more hemodynamic support up to 6.2 L/min, but a surgical cutdown for implantation is required. The DanGer shock trial showed a lower 180-day mortality in STEMI-related cardiogenic shock when compared to standard of care [4], but overall data on its effect on patient outcome are limited and device-related complications are frequent [5–7].

Since 2020, Impella CP support has been employed at the University Medical Centres of Groningen and Utrecht. Both centres are tertiary referral hospitals for advanced heart failure therapies and maintain dedicated durable left ventricular assist device (LVAD) programmes. Previously, we have shown a low 30-day mortality rate in patients supported with veno arterial extracorporeal membrane (VA-ECMO) in combination with Impella [8]. In certain circumstances, Impella-only support is chosen when cardiogenic shock is less severe or when VA-ECMO is deemed too invasive. For the current study, we aimed to explore in which way Impella-only support can function as bridge-to-recovery or bridge-to-decision for further advanced heart failure treatment.

## Methods

### Study population

This observational study included all consecutive patients who were treated with an Impella CP for cardiogenic shock in the University Medical Centres of Groningen and Utrecht. Patients treated between December 2020 and August 2023 were enrolled. Use of additional mechanical support was an exclusion criterium as well as elective high-risk PCI. Severity of shock was classified using the SCAI shock stage [9]. Indication for Impella CP use was decided by a multidisciplinary team, consisting of a (advanced) heart failure cardiologist, an interventional cardiologist and an intensivist, usually supported by a cardiothoracic surgeon and a cardiac anaesthesiologist. Regarding the selection of mechanical circulatory support modality, there is no pre-established protocol. Decisions are based on clinical parameters (pulmonary oedema and end-organ perfusion), echocardiographic parameters (LV and RV function and dimensions, spontaneous contrast, and aortic valve opening) and haemodynamic parameters (for example, right heart catheterisation, if performed). During this discussion, we also consider whether a patient is a suitable candidate for LVAD or heart transplantation. All data were collected from our electronic patient records. Our institution waived the need for patient consent due to the study’s retrospective design, in which individual patients were not identified.

### Outcomes

The primary endpoint was all-cause mortality at 30 days. Secondary endpoints encompassed LVAD implantation, cardiac recovery (defined as successful durable weaning of Impella CP without the need for repeated mechanical support within 30 days), and safety outcomes, including TIMI major bleeding (defined as intracranial bleeding or a haemoglobin drop exceeding 3.0 mmol/l), haemolysis (indicated by lactate dehydrogenase levels of ≥ 1000 U/l in conjunction with two distinct haptoglobin levels < 0.3 g/L, as previously outlined) [10], vascular access complications, device failure necessitating extraction, and the initiation of renal replacement therapy. Vascular access complications were categorised as either ischaemic (requiring intervention for limb ischaemia) or bleeding (requiring intervention). For subgroup analysis, patients were categorised as Impella-limited (i.e. Impella CP support because escalation to VA-ECMO and/or LVAD therapy is considered a bridge too far at that moment because of comorbidities) or as Impella-optimal (i.e. Impella CP support is sufficient).

### Statistical analysis

Statistical analyses were performed with IBM SPSS Statistics, version 20.0.1 (IBM Corp). Normally distributed variables were reported as mean and standard deviation and were statistically tested with the Student’s t‑test. Non-normally distributed variables were presented as median and interquartile range (IQR). Mann-Whitney U test was used for comparison between groups. Percentages were compared using χ^2^ test.

## Results

### Baseline characteristics

A total of 28 patients were included in our analysis (Tab. 1). Both centres implanted 14 Impella CP devices. Mean age was 60 (± 13) years, and most patients were male (71%) and of Caucasian descent (89%). Mean BMI was 24.5 (± 4.5) kg/m^2^.

**Table 1:** Baseline characteristics of the total population and divided in the Impella-limited and Impella-optimal groups

Baseline characteristics	Total (*n* = 28)	Limited (*n* = 10)	Optimal (*n* = 18)	*p*-value
Age (years)	60 (13)	69 (11)	55 (11)	< 0.001
Gender (male, %)	20 (71)	8 (80)	12 (67)	0.45
BMI (kg/m^2^)	24.5 (4.5)	26.1 (5.7)	23.7 (3.6)	0.19
Race (caucasian, %)	25 (89)	9 (90)	16 (89)	0.93
Hypertension (*n*, %)	7 (25)	4 (40)	3 (17)	0.17
Diabetes mellitus (*n*, %)	3 (11)	0 (0)	3 (17)	0.17
Peripheral artery disease (*n*, %)	1 (4)	1 (10)	0 (0)	0.17
Ischemic CVA (*n*, %)	0 (0)	0 (0)	0 (0)	n/a
Myocardial infarction (*n*, %)	9 (32)	3 (30)	6 (33)	0.86
Percutaneous revascularisation (*n*, %)	5 (18)	3 (30)	2 (11)	0.21
*Smoking* (*n*, %)*				0.28
– Never smoker	14 (54)	6 (67)	8 (47)	
– Previous smoker	4 (155/28)	2 (22)	2 (12)	
– Current smoker	8 (31)	1 (11)	7 (41)	
Atrial fibrillation (*n*, %)	3 (11)	1 (10)	2 (11)	0.93
Chronic kidney disease (*n*, %)	3 (11)	1 (10)	2 (11)	0.93
ICD/CRT‑D (*n*, %)	5 (18)	2 (20)	3 (17)	0.83
Pacemaker (*n*, %)	0 (0)	0 (0)	0 (0)	n/a

*Missing data for 2 patients

### Clinical profile of cardiogenic shock

At the time of Impella insertion, patients were in overt cardiogenic shock, with a mean heart rate of 103 (± 23) per minute, a mean systolic blood pressure of 87 (± 15) mm Hg and a diastolic blood pressure of 60 (± 11) mm Hg (Tab. 2). Median lactate was 2.8 mmol/l (IQR 2.0–3.6). Inotropes and vasopressors were used in 86 and 75% of the cases, respectively. Nine patients (32%) were on invasive mechanical ventilation at presentation. Five patients (8%) received cardiopulmonary resuscitation prior to implantation. Acute coronary syndrome was present in 13 patients (46%), acute-on-chronic heart failure in 12 patients (43%) and acute cardiogenic shock in 3 patients (due to cardiac sarcoidosis presenting with ventricular tachycardia, giant cell myocarditis and COVID-19 myocarditis). Median Impella CP support duration was 63 h (IQR 29–95).

**Table 2:** Clinical profile of cardiogenic shock during Impella implantation of the total population and divided in the Impella-limited and Impella-optimal groups

Clinical profile of cardiogenic shock	Total (*n* = 28)	Limited (*n* = 10)	Optimal (*n* = 18)	*p*-value
Heart rate (/min)	103 (23)	100 (20)	104 (25)	0.60
Systolic blood pressure (mm Hg)	87 (15)	83 (19)	88 (13)	0.05
Diastolic blood pressure (mm Hg)	60 (11)	53 (9)	63 (11)	0.78
Kreatinine (umol/l)	108 (88–154)	133 (88–183)	102 (87–140)	0.88
Sodium (mmol/l)	137 (6)	136 (7)	137 (5)	0.14
Potassium (mmol/l)	4.1 (1.1)	4.6 (1.3)	3.9 (0.9)	0.27
Ureum (mmol/l)	10.8 (6.9–20.0)	7.3 (6.2–23.3)	12.2 (7.0–20.1)	
Hb (mmol/l)	8.2 (7.2–8.8)	8.7 (8.2–9.4)	7.5 (7.2–8.8)	
Leucocytes (× 10^9^/l)	13.2 (8.9–17.6)	15.8 (8.6–22.4)	11.3 (8.8–16.8)	
Lactate (mmol/l)	2.8 (2.0–3.6)	3.4 (2.3–5.3)	2.7 (1.8–3.1)	0.23
Bilirubine (umol/L)	16 (10.3–20.8)	18 (9.5–38)	13 (9–18.5)	
CRP (mg/dl)	51 (13–81)	48 (13–178)	51 (13–104)	
Trombocytes (× 10^9^/l)	240 (161–338)	248 (104–385)	231 (176–319)	
Inotropes use (*n*, %)	24 (86)	8 (80)	16 (89)	0.52
Vasopressur use (*n*, %)	21 (75)	8 (80)	13 (72)	0.65
Mechanical ventilation (*n*, %)	9 (32)	4 (40)	5 (28)	0.64
Cardiopulmonary resuscitation (*n*, %)	5 (18)	2 (20)	3 (17)	0.83
*SCAI shock classification (n, %)*				0.23
– Stage C (*n*, %)	5 (18)	1 (10)	4 (22)	
– Stage D (*n*, %)	20 (71)	9 (90)	11 (61)	
– Stage E (*n*, %)	3 (11)	0 (0)	3 (17)	
*Underlying cardiomyopathy (n, %)*				0.23
– Ischaemic cardiomyopathy (*n*, %)	21 (75)	9 (90)	12 (67)	
– Genetic cardiomyopathy (*n*, %)	2 (7)	0 (0)	2 (11)	
– Inflammatory (*n*, %)	3 (11)	0 (0)	3 (17)	
– Cardiomyopathy unknown etiology (*n*, %)	1 (4)	1 (0)	0 (0)	
– Toxic cardiomyopathy (*n*, %)	1 (4)	0 (0)	1 (6)	
*Cause of cardiogenic shock (n, %)*				0.25
– Acute coronary syndrome		7 (70)	6 (33)	
– Acute failure with known cardiomyopathy	12 (43)	3 (30)	9 (50)	
– Ventricular tachycardia	1 (4)	0 (0)	1 (6)	
– Acute myocarditis	2 (7)	0 (0)	2 (11)	
Access site, left femoral artery (*n*, %)	6 (21)	4 (40)	2 (11)	0.07
Duration of Impella support (h)	63 (29–95)	34 (27–71)	72 (41–117)	0.09

### Clinical outcomes

Thirty-day mortality was observed in 13 patients (46%) (Tab. 3). LVAD implantation was performed in 10 patients (36%). Full cardiac recovery, without further therapy escalation was observed in 5 patients (17%). Table S1 (Electronic Supplementary Material) provides full details of each patient. Eight patients (29%) died due to ongoing refractory shock, and one as a result of refractory ventricular arrhythmias (4%). One patient died after LVAD implantation because of right-sided heart failure. Four patients died due to septic shock (14%). Both refractory pulmonary failure and uncontrollable gastrointestinal bleeding were cause of death in one case.

**Table 3:** Clinical Outcomes and safety and complications of the total population and divided in the Impella-limited and Impella-optimal groups

	Total (*n* = 28)	Limited (*n* = 10)	Optimal (*n* = 18)	*p*-value
*Clinical outcomes*				
LVAD implantation (*n*, %)	10 (36)	1 (10)	9 (50)	0.03
Heart transplantation (*n*, %)	0 (0)	0 (0)	0 (0)	n/a
30-day cardiac recovery (*n*, %)	5 (18)	0 (0)	5 (39)	0.02
30-day mortality (*n*, %)	13 (46)	10 (100)	3 (17)	< 0.001
*Safety/complications*				
(i)CVA (*n*, %)	0 (0)	0 (0)	0 (0)	n/a
Vascular (*n*, %)	3 (11)	2 (20)	1 (6)	0.10
– Bleeding, requiring intervention	2 (7)	2 (20)	0 (0)	
– Ischemic, requiring intervention	1 (4)	0 (0)	1 (6)	
Hemolysis (*n*, %)	4 (14)	1 (10)	3 (17)	0.63
Major bleeding (*n*, %)	2 (7)	0 (0)	2 (11)	0.26
Need for blood transfusion (*n*, %)	9 (32)	3 (30)	6 (35)	0.78
Acute kidney injury (*n*, %)	7 (25)	3 (30)	4 (24)	0.71
Impella failure requiring extraction (*n*, %)	2 (7)	1 (10)	1 (6)	0.65

### Safety and complications

Vascular complications were observed in three patients (11%): one ischaemic event leading to critical limb ischaemia and the necessitating to extraction of the Impella CP (without the need of limb amputation), and two bleeding complications (Tab. 3). Major bleeding was seen in two patients (7%). Haemolysis was present in four patients (14%) and led to extraction of the Impella CP device in one case. In one case, Impella CP failure due to thrombosis was observed, requiring extraction. Acute kidney injury (i.e. initiation of renal replacement therapy) was present in 7 patients (25%). No differences between the Impella-optimal and Impella-limited group were observed.

### Impella-limited versus Impella-optimal

Ten patients (36%) were classified as Impella-limited (due to liver failure, suspicion of COVID-19 pneumonia, advanced age, frailty and a neurodegenerative disease [Tab. S1, Electronic Supplementary Material]). Patients in the Impella-limited group were significantly older (69 vs. 55 years, *p* < 0.001) and the systolic blood pressure tended to be lower (83 versus 88 mm Hg, *p* = 0.05). In the Impella-limited group, LVAD implantations were less often performed (10 vs. 50%, *p* = 0.03), 30-day mortality was significantly higher (100 vs. 17%; *p* < 0.001) and 30-day recovery was lower (0 vs. 39%, *p* = 0.02).

## Discussion

This case series describes the first experience with Impella-only support in acute cardiogenic shock in two large tertiary hospitals in the Netherlands. Overall, 30-day mortality was 46% and LVAD implantation was performed in 36% of the patients. Differences were observed between the Impella-limited and the Impella-optimal group (i.e. Impella-limited support because escalation to VA-ECMO and/or LVAD therapy was considered a bridge too far versus Impella-optimal support deemed sufficient): in the Impella-optimal group, LVAD implantation was performed in 50% (versus 10%) and 30-day mortality was only 17% (versus 100%). Although the numbers are limited, the current data can provide insight into the opportunities and limitations of Impella-driven mechanical circulatory support in critically ill patients in the Netherlands.

### 30-day mortality

The current study showed a high 30-day mortality of 46%, which is comparable to previously reported [11]. Despite revascularisation and mechanical support, the mortality of cardiogenic shock still remains high [10]. This is of interest because the role of Impella CP in cardiogenic shock is still under debate; however, the results of the DanGer Shock trial showed a mortality benefit in STEMI-related cardiogenic shock [4]. Nevertheless, data are conflicting and patient groups remain very heterogeneous. In the IMPRESS in Severe Shock trial, Impella and IABP-supported patients had similar 30-day survival rates [12]. Meta-analyses showed equal mortality rates in patients with cardiogenic shock and supported with either Impella or IABP [6, 11]. However, these data suggest that Impella might be less favourable in older patients with multi-organ failure. In our study, in the Impella-limited group, the mortality rate was 100%. In the Impella-optimal group, LVAD implantation was performed in 50% and cardiac recovery was 39%. 30-day mortality was 17%. These results underline that adequate patient selection is key to successful mechanical support.

### Advanced heart failure therapy

#### Impella CP as a bridge-to-therapy

The decision to escalate to mechanical support therapy often has to be made within a (very) short time. Our data underline that, in case of timely and adequate selection, certain patients can benefit from Impella-only support. In the Impella-limited group, Impella CP support was inadequate and more cardiac support was indicated, or palliative care should have been pursued. These data underline the importance of careful and thorough screening of cardiogenic shock patients, in particular in the setting of potential LVAD candidacy. Because of the high impact of LVAD therapy on the patient’s quality of life, informed consent is of the utmost importance. In refractory cardiogenic shock, temporary mechanical support can aid in buying time to inform patients and their families, to investigate patients’ preferences regarding treatment options, and to wait for the optimal timing of implantation of an LVAD or heart transplantation.

### Future perspectives

Effective management of refractory cardiogenic shock using temporary mechanical circulatory support will remain a challenge. Focusing on better patient selection and ensuring appropriate mechanical circulatory support will hopefully improve future outcomes in cardiogenic shock. A centre-specific cardiogenic shock team could aid in this decision process [13]. The ULYSS (NCT05366452) will shed new light on this topic [14].

### Limitations

Our study is limited by selection bias due to its retrospective design, and generalisability could be limited because patients from two academic centres were used. Furthermore, it is difficult to draw definitive conclusions regarding Impella CP support in cardiogenic shock due to the small number of patients included and the heterogeneity of the population. A national prospective study of patients treated with Impella CP support is being prepared.

## Conclusions

This retrospective cohort study suggests that Impella CP-only support in cardiogenic shock can be feasible and safe as a bridge-to-recovery or as a bridge-to-decision to assess candidacy for potential LVAD implantation in selected cases. If a decision has been made not to escalate treatment to advanced mechanical circulatory support, LVAD implantation, or heart transplantation, the 30-day mortality rate remains 100% despite the use of Impella CP support. Patient selection in mechanical circulatory support is key to success.

## Supplementary Information

**Table S1. **Detailed clinical profile of patients supported with Impella.
