# Supplementary material for: Microaxial flow pump in cardiogenic shock: a retrospective cohort study on outcomes and feasibility as a bridge to LVAD implantation
Source: Neth Heart J. 2025 Jul 3;33(7-8):226–31. doi: 10.1007/s12471-025-01963-2 (PMC12274170; doi:10.1007/s12471-025-01963-2)
Supplement: Supplementary file 1 — Table S1. Detailed clinical profile of patients supported with Impella. [file 12471_2025_1963_MOESM1_ESM.docx]

| **Case** | **Age** | **Gender** | **Cardiomyopathy** | **Cause** | **SCAI** | **Bridge to** | **Impella-only**  **because** | **LVAD implantation** | **Duration**  **support** | **Complications** | **Outcome 30-day** |
| --- | --- | --- | --- | --- | --- | --- | --- | --- | --- | --- | --- |
| 1 | 81 | male | ischaemic | ACS | D | recovery | age | no, high age | 48h | minor bleeding | died, CS |
| 2 | 43 | male | unknown | chronic | D | decision | liver failure | no, multi-organ failure  right heart failure | 28h | hemolysis | died, CS |
| 3 | 66 | male | ischaemic | ACS | D | decision | possible COVID-19  pneumonia | yes | 24h | none | died, septic shock |
| 12 | 64 | male | ischaemic | ACS | D | decision | Neurodegenerative disease | no, patient’s request and neurodegeneratieve disease | 70h | minor bleeding | died, CS |
| 9 | 61 | male | ischaemic | ACS | D | decision | on patient’s request | no, patient’s request | 34h | critical limb ischaemic | died, CS |
| 15 | 73 | female | ischaemic | ACS | D | recovery | Frailty | no, frailty | 34h | none | died, CS |
| 17 | 70 | male | ischaemic | chronic | C | recovery | Frailty | no, frailty | 80h | none | died, CS |
| 19 | 77 | female | ischaemic | ACS | D | recovery | Age | no, high age | 10h | none | died, VT’s |
| 22 | 76 | male | ischaemic | ACS | D | recovery | Age | no, high age | 32h | Minor bleeding | died, CS |
| 25 | 75 | male | ischaemic | chronic | D | recovery | Age | no, high age | 74h | none | died, septic shock |
| 4 | 64 | female | sarcoidosis | VTs | D | decision | ECMO not necessary | yes | 96h | none | died, right heart failure |
| 5 | 42 | male | genetic | chronic | D | decision | ECMO not necessary | yes | 64h | hemolysis | alive |
| 6 | 54 | male | genetic | chronic | D | decision | ECMO not necessary | yes | 12h | none | alive |
| 11 | 62 | male | ischaemic | chronic | D | decision | ECMO not necessary | yes | 120h | none | alive |
| 14 | 64 | female | toxic | chronic | C | decision | ECMO not necessary | yes | 61h | none | alive |
| 16 | 47 | male | iv | chronic | D | decision | ECMO not necessary | yes | 116h | Hemolysis, minor bleeding | alive |
| 20 | 48 | male | ischaemic | chronic | D | decision | ECMO not necessary | yes | 21h | none | alive |
| 26 | 48 | male | ischaemic | chronic | D | decision | ECMO not necessary | yes | 164h | none | alive |
| 27 | 48 | male | ischaemic | ACS | D | decision | ECMO not necessary | yes | 21h | none | alive |
| 7 | 63 | male | ischaemic | ACS | C | recovery | ECMO not necessary | no, not necessary | 90h | bleeding | alive |
| 8 | 40 | male | ischaemic | ACS | D | recovery | ECMO not necessary | no, not necessary | 306h | hemolysis | alive |
| 18 | 75 | female | ischaemic | ACS | E | recovery | ECMO not necessary | no, not necessary | 7h | Retrosternal hematoma | alive |
| 23 | 53 | female | ischaemic | ACS | D | Bridge to MVR | ECMO not necessary | no, not necessary | 66h | None | alive |
| 28 | 32 | female | myocarditis | Acute | E | recovery | ECMO not necessary | no, not necessary | 96h | None | alive |
| 21 | 57 | male | ischaemic | ACS | E | decision | ECMO not necessary | no, not necessary | 48h | none | Alive |
| 24 | 58 | female | myocarditis | acute | C | recovery | ECMO not necessary | no, not necessary | 195h | Minor bleeding | Alive |
| 13 | 69 | male | ischaemic | chronic | D | recovery | ECMO not necessary | no, not necessary | 48h | major bleeding, thrombosis Impella | died, pneumonia, melena. |
| 10 | 62 | male | ischaemic | chronic | C | decision | ECMO not necessary | no, patient’s request | 78h | major bleeding | died, CS |

**Table S1.** Detailed clinical profile of patients supported with Impella.
